# Psychological, behavioural and biological factors associated with gastrointestinal symptoms in autistic adults and adults with autistic traits

**DOI:** 10.1177/13623613231155324

**Published:** 2023-02-16

**Authors:** EB Warreman, LA Nooteboom, MB Terry, HW Hoek, PJM Leenen, EFC van Rossum, D Ramlal, RRJM Vermeiren, WA Ester

**Affiliations:** 1 Leiden University Medical Center, The Netherlands; 2 Columbia University, USA; 3 University Medical Center Groningen, The Netherlands; 4 Parnassia Group, The Netherlands; 5 Erasmus University Medical Center, The Netherlands

**Keywords:** adults, autism spectrum disorders, autistic traits, gastrointestinal, psychosomatic

## Abstract

Gastrointestinal symptoms and their relation to physical and mental aspects in adults with an autism spectrum disorder (ASD) are poorly understood, despite their high prevalence. Therefore, the aim of this study is to examine psychological, behavioural and biological factors associated with gastrointestinal symptoms in adults with ASD (traits). We included 31,185 adults from the Lifelines Study. Using multivariable logistic regression, we analysed the association between gastrointestinal symptoms and psychological, behavioural (questionnaire-assessed) and physically measured biological factors in adults with ASD (*n* = 309), without ASD (*n* = 30,876), and in the quartiles with highest (*n* = 7783) and lowest (*n* = 7783) Autism Spectrum Quotient-10 sum scores. In the ASD-group, gastrointestinal symptoms were associated with psychiatric comorbidity (odds ratio: 2.71, 95% confidence interval: 1.51–4.85), more stress (odds ratio: 1.15, 95% confidence interval: 1.06–1.26), and worse perceived health (odds ratio: 2.32, 95% confidence interval: 1.62–3.34). In the quartile with the highest Autism Spectrum Quotient-10 sum scores, gastrointestinal symptoms were also associated with these psychological factors, and with less physical activity (odds ratio: 0.95, 95% confidence interval: 0.92–0.98). Our study demonstrates that not only adults with ASD but also adults with autistic traits are at increased risk for gastrointestinal symptoms, which is associated with psychological and behavioural factors. This suggests that an integrated psychosomatic approach of gastrointestinal symptoms in adults with ASD (traits) is needed.

**Lay abstract:**

Little is known about factors related to the increased risk for gastrointestinal symptoms in adults with an autism spectrum disorder (ASD), while the negative impact of gastrointestinal symptoms is evident. Especially, the relationship between gastrointestinal symptoms and psychological, behavioural, and biological risk factors in adults with ASD (traits) is unclear. Autistic peer support workers and autism-advocates also emphasised the importance of identifying risk factors, because of the high prevalence of gastrointestinal problems in people with ASD. Therefore, our study investigated which psychological, behavioural, and biological factors are associated with gastrointestinal symptoms in adults with ASD or with autistic traits. We analysed data from 31,185 adults in the Dutch Lifelines Study. Questionnaires were used to evaluate the presence of an autism spectrum disorder diagnosis, autistic traits, gastrointestinal symptoms, psychological and behavioural factors. Biological factors were examined with body measurements. We found that not only adults with ASD but also adults with higher levels of autistic traits were at increased risk for gastrointestinal symptoms. Adults with ASD who experienced psychological problems (psychiatric problems, worse perceived health, chronic stress) had a higher risk for gastrointestinal symptoms than adults with ASD without these psychological problems. Moreover, adults with higher levels of autistic traits were less physically active, which was also associated with gastrointestinal symptoms. In conclusion, our study highlights the relevance of identifying psychological problems and evaluating physical activity when trying to help adults with ASD or autistic traits and gastrointestinal symptoms. This suggests that healthcare professionals should be more aware of behavioural and psychological risk factors when evaluating gastrointestinal symptoms in adults with ASD (traits).

## Introduction

Gastrointestinal (GI) problems are more prevalent in people with an autism spectrum disorder (ASD) than in the general population (Croen et al., 2015; Hand et al., 2020; McElhanon et al., 2014). However, previous studies concerning GI symptoms mainly included children with ASD (Holingue et al., 2018; Lefter et al., 2019; McElhanon et al., 2014). Consequently, in-depth knowledge about GI-symptoms in autistic adults is lacking. A case-control study reported a prevalence of 35% GI disorders in adults with ASD (mean age 29 years old) compared to 28% in adults without ASD (Croen et al., 2015). In older adults with ASD (65+ years old), GI disorders seem to be more common than in non-autistic adults (49% vs 25%), as are other GI conditions such as gastroenteritis or constipation (Hand et al., 2020). It is also known that the risk for GI disorders is increased in adults with both ASD and intellectual disability (Bishop-Fitzpatrick & Rubenstein, 2019; Gilmore et al., 2021). However, to our knowledge, there are no previous studies investigating other factors (e.g. psychological, behavioural and biological) associated with GI symptoms in adults with ASD. Thus, comprehensive knowledge of GI symptoms in autistic adults is limited, while a better understanding of their increased risk for GI problems is needed (Leader et al., 2021). In this study, we investigated the presence of GI symptoms, rather than diagnosed GI disorders, because of the high prevalence of functional GI problems in a broader sense in adults with ASD (Bishop-Fitzpatrick & Rubenstein, 2019; Croen et al., 2015).

When investigating factors associated with GI symptoms, psychological factors such as stress, anxiety and depression should be taken into account. Stress is a mediator in GI-symptoms, as stress influences both gut function and microbiota composition through the hypothalamic-pituitary-adrenal (HPA) axis, and reduces gut motility via the noradrenergic system (Cacioppo et al., 2007; De Palma et al., 2014). The individual’s immune status is also closely intertwined with HPA axis activity, personal stress levels, the gastrointestinal condition and microbiota (Hollins & Hodgson, 2019). Relevant immunological markers that are altered by stress are for example C-reactive protein (CRP) and leukocyte counts (Del Giudice & Gangestad, 2018; Dijkstra-de Neijs et al., 2020). In addition, in children with ASD, a positive association between the concentration of the stress hormone cortisol and lower GI-tract symptoms was found (Ferguson et al., 2016). Moreover, stress is related to psychiatric symptoms, such as depression and anxiety (Tafet & Nemeroff, 2016). People with ASD often experience high levels of stress (Baron et al., 2006; Bishop-Fitzpatrick et al., 2015; Hirvikoski & Blomqvist, 2015) and psychiatric comorbidities are very common (Hand et al., 2020; Lai et al., 2014). Also, anxiety has been associated with GI problems in children with ASD (Ferguson et al., 2017).

Furthermore, behavioural factors, such as physical (in)activity and alcohol abuse, can have an impact on GI symptoms through the gut-brain axis (Bilski et al., 2018; Chen & Haber, 2021). It is also known that children with ASD are more susceptible for food selectivity and insufficient oral intake, which can affect the intestinal microbiome (Bandini et al., 2010; Murtaza et al., 2017).

Next to the above-mentioned immunological markers that can be altered in relation to stress and GI symptoms, other biological factors that are of concern when investigating GI symptoms are body mass index (BMI) and waist circumference (to measure abdominal obesity). Increased BMI and abdominal obesity are associated with GI problems, such as irritable bowel syndrome (IBS) and gastroesophageal reflux (Emerenziani et al., 2019; Foxx-Orenstein, 2010; Lee et al., 2015).

In summary, psychological, behavioural and biological factors affecting the gut-brain axis should be taken into account when researching GI symptoms in autistic adults.

While adults with ASD tend to have more GI symptoms than the general population, an integrated approach analysing psychological, behavioural, and biological factors associated with GI symptoms in autistic adults is missing. A better understanding of these factors and their putative associations in adults with ASD is relevant to optimise the prevention and treatment of these GI symptoms. The underlying theory entails that autistic traits and related factors, such as stress, depression, anxiety, sensory dysregulation and food selectivity contribute to GI symptoms (Amos et al., 2019; Harris et al., 2021; Mazurek et al., 2013; Williams & Gotham, 2022). Therefore, the main goal of this study is to use an integrated approach to explore which psychological, behavioural and biological factors are associated with GI symptoms in adults with ASD and in adults with autistic traits. In addition, our study leads to more insights regarding psychological, behavioural and biological characteristics in both adults with ASD and in the general adult population with autistic traits.

## Methods

### Study population

All data were extracted from the Lifelines database. Lifelines is a multi-disciplinary prospective population-based cohort study examining in a unique three-generation design the health and health-related behaviours of 167,729 persons living in the north of the Netherlands. It employs a broad range of investigative procedures in assessing the biomedical, socio-demographic, behavioural, biological and psychological factors which contribute to the health and disease of the general population, with a special focus on multi-morbidity and complex genetics (Scholtens et al., 2015). Baseline assessment took place between 2007 and 2013, and the second assessment was performed from 2014 until 2017. Subsequently, in 2019, an autism questionnaire (AUTQ) was sent to 109,352 Lifelines participants. The AUTQ consisted of the short version of the Autism Spectrum Quotient (AQ-10) and a self-report question regarding the presence of an ASD diagnosis. Thus, to be eligible for this study, participants had to be able to submit self-report surveys.

**Figure 1.**
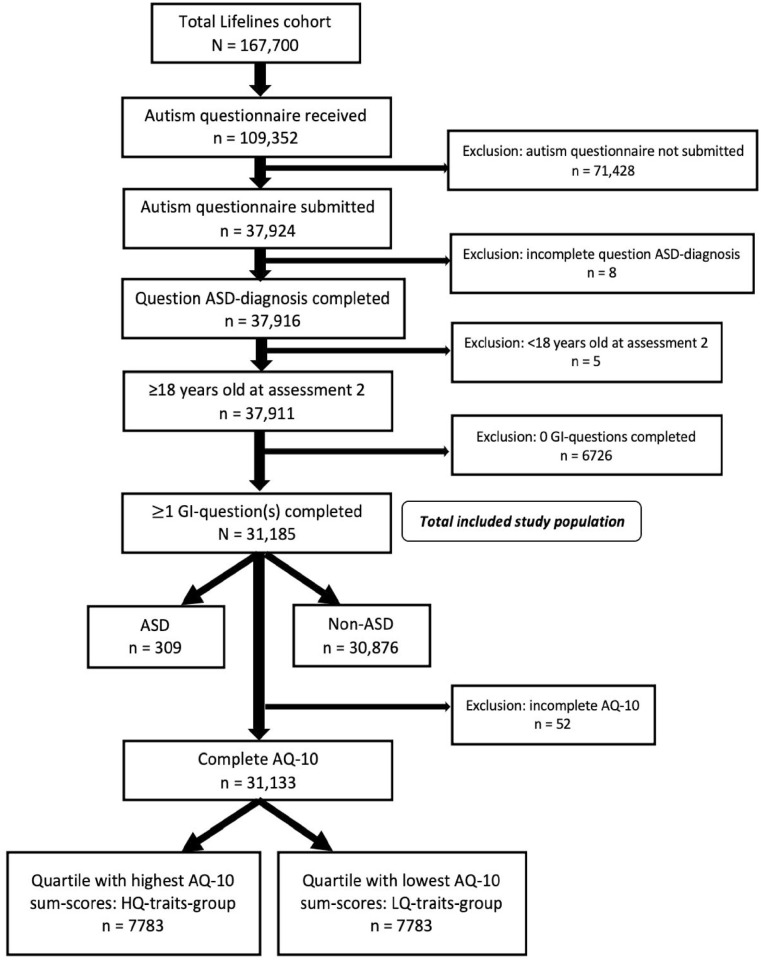
Flow diagram of study population.

We included 31,185 participants (Figure 1), aged 18 years or older at the start of the second Lifelines assessment, who reported the presence or absence of an ASD diagnosis, and provided any answer to one or more questions regarding GI symptoms in the respective questionnaire (see below). These 31,185 included participants were first divided into two groups: ASD (*n* = 309) and non-ASD (*n* = 30,876). Next, for formation of groups based on AQ-10 sum scores, cases with an incomplete AQ-10 were excluded from the whole included population (Figure 1). The remaining participants were categorised in quartiles, based on their AQ-10 sum scores. The highest quartile (HQ-traits-group: *n* = 7783), consisting of 25% of participants with the highest AQ-10 sum scores, and the lowest quartile (LQ-traits-group: *n* = 7783), consisting of 25% of participants with the lowest AQ-10 sum scores, were used for analyses.

### Measures

#### Autistic traits

The AQ-10 is an instrument to quantify the degree of autistic traits in adults with average intelligence (Allison et al., 2012). It should not be used to predict an ASD diagnosis, since studies found varying sensitivity and specificity values of 62%–80% and 28%–87%, respectively (Ashwood et al., 2016; Booth et al., 2013; Sizoo et al., 2015). However, the AQ-10 is indeed a valid measure in epidemiological studies to grade the level of autistic symptoms in adult participants (Lundin et al., 2019; Warrier et al., 2020).

#### Gastrointestinal symptoms

**Figure 2.**
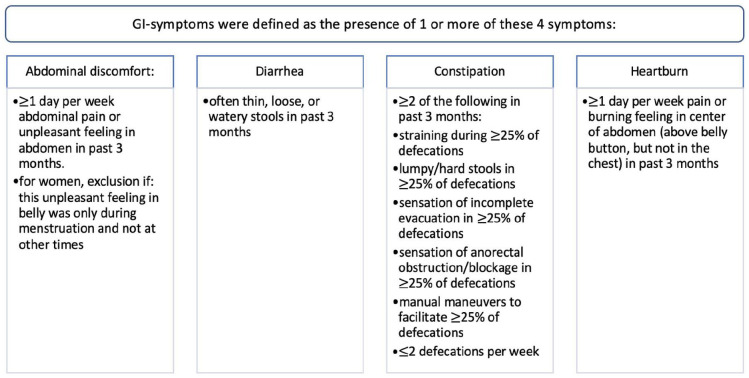
Definition of gastrointestinal symptoms.

The Rome III IBS Diagnostic Questionnaire addresses different variables concerning GI-symptoms (Thompson et al., 2006). In our study, we defined GI symptoms as the self-report of one or more of the following complaints: abdominal discomfort/pain, diarrhoea, constipation and/or heartburn. The presence of each symptom was rated on a 5- or 7-point Likert-type-scale. Cut-off points (described in Figure 2) were based on clinical relevance and definitions of functional bowel disorders (Longstreth et al., 2006; Palsson et al., 2016). As supplementary analysis, the prevalence of GI diseases (Crohn’s disease, ulcerative colitis, coeliac disease, gastric ulcer and/or gallstones) was compared between adults with and without ASD.

#### Psychological factors

Psychiatric comorbidity was defined as the presence of one or more of the following diagnoses: any current depressive disorder (major depressive disorder, dysthymia) and/or any current anxiety disorder (panic disorder, agoraphobia, social phobia, generalised anxiety disorder), as determined in a face-to-face mini international neuropsychiatric interview (MINI; based on the *DSM*-IV-TR). Another psychological measure included self-reported stress, using the long-term difficulties inventory (LDI). LDI sum scores were calculated to compare stress levels between groups. We assessed perceived health with a RAND-question about general health as a continuous variable, asking participants to answer on a 5-point Likert-type scale: ‘How would you rate your health generally speaking?’

#### Behavioural factors

Physical activity was measured using the following question from the Short Questionnaire to Assess Health-enhancing physical activity (SQUASH): ‘Adding everything up, on how many days per week on average are you involved in cycling, doing odd jobs, gardening, sport, or other strenuous activities for at least 30 minutes?’. Alcohol use was assessed with this question from the Flower Food Frequency questionnaire (FFQ): ‘During the past month, how many glasses of alcoholic drinks did you drink per day on average?’. For this variable, the cut-off for binary analyses was set at an average alcohol intake of more than two glasses per day (Rausch et al., 2022; Wouters et al., 2020).

#### Biological factors

Waist circumference and height and weight (for calculation of BMI) were measured by trained Lifelines’ personnel during a physical visit of the second assessment. In addition to the level of psychological stress as determined by the LDI, we evaluated counts of different types of leukocytes and CRP levels as measures of inflammation and therefore indirect physical components of perceived stress (Del Giudice & Gangestad, 2018; Dijkstra-de Neijs et al., 2020). Blood samples were also drawn during a physical visit of the second assessment. CRP levels were processed using a nephelometric assay in a Roche Modular analyser (Roche, Basel, Switzerland).

#### Covariates

Socioeconomic status was determined with self-reported employment status and educational attainment. Employment status was a dichotomous question on whether the participant, at that moment, did paid work for one or more hours per week. Educational attainment was categorised as low (no education, primary, lower or preparatory vocational education or lower general secondary education), middle (intermediate vocational education or apprenticeship, higher general secondary education or pre-university secondary education), high (higher vocational education or university) or other.

#### Autistic community involvement

The research team was advised by members of the Dutch Academic Workplace for Autism’ (including autistic adults and clinicians) about the research question, selection of relevant variables and interpretation of the results.

### Statistical analyses

We used IBM SPSS Statistics version 25 for all data analyses. Before we performed our main analyses, we executed non-response analyses. The goal of the first non-response analysis was to map characteristics of the 71,428 Lifelines’ participants that did receive the AUTQ, but did not submit this questionnaire (and could therefore not be included in our study). The second non-response analysis was performed to compare the total included study population (*N* = 31,185) with the participants that were excluded because of an incomplete GI questionnaire (*n* = 6726).

Then, we first analysed basic characteristics with univariable analyses comparing the ASD- versus non-ASD-groups, and the HQ-traits-group versus LQ-traits-group. After that, psychological, behavioural and biological factors were also compared for the ASD- versus non-ASD-group and the HQ-traits-group versus LQ-traits-group, with univariable and multivariable analyses. In univariable analyses, categorical variables were analysed with Chi-square tests or Fisher’s exact tests, and continuous variables with Student *t* tests or Mann–Whitney U tests. *P* values below 0.05 were considered statistically significant. Correction for age, sex, and socioeconomic status was carried out with multivariable logistic regression for categorical variables and multivariable linear regression for continuous variables.

Next, multivariable logistic regression with the presence of (one or more) GI symptoms as an outcome measure was performed. These logistic regression models were executed for each of the included psychological behavioural, and biological factors in the ASD-, non-ASD-, HQ-traits- and LQ-traits-groups. Age, sex and socioeconomic status were included as confounders in multiple logistic regression (Bytzer et al., 2001; Knutsson & Bøggild, 2010). Transformation of skewed data was not indicated, because the assumptions of logistic regression were met based on the nature of the distributions. In these logistic regression models, exclusion of participants with ASD from the HQ-traits-group did not result in different outcomes. Therefore, these participants were not excluded from the HQ-traits-group.

Primary interaction term analysis was executed in the total included study population (*N* = 31,185), to identify the significance of the interaction effect of an ASD diagnosis for each investigated psychosocial, behavioural and biological factors with GI symptoms as dependent variable. Secondary interaction analysis was performed in the combined HQ-traits-group and LQ-traits-group (*n* = 15,566), to identify the significance of the interaction effect of reporting the highest levels of autistic traits (HQ-traits-group).

Imputation of missing data was not needed because the prevalence of missing data did not differ between ASD and the control group, and the origin of these missing data was regarded as random. CRP levels were not analysed in the ASD-group since these data were only available for 3 participants in this group. This was due to the fact that from the total Lifelines cohort (*N* = 167,700), only in approximately 500 participants CRP levels were determined during the second assessment.

### Ethical approval

Ethical approval for the Lifelines study (reference number NL17981.042.07) was obtained from the medical ethical committee of the University Medical Centre Groningen, The Netherlands.

## Results

### Basic characteristics

In the total study population of 31,185 adults, 1% (*n* = 309) reported an ASD diagnosis (Table 1). Of these 309 adults with ASD, 86.4% (*n* = 267) was categorised in the HQ-traits-group (i.e. group with highest AQ-10 sum scores). Moreover, in adults with ASD, the median AQ-10 sum score of 6.0 (interquartile range (IQR) 4.5–7.5) was 4.0 points higher than in adults without reported ASD (*p* < 0.01). Socioeconomic status was lower in adults with ASD compared with adults without ASD, and in the HQ-traits-group compared with the LQ-traits-group.

**Table 1.** Basic characteristics: ASD- versus non-ASD-group and HQ-traits- versus LQ-traits-group.

	ASD, *n* = 309		non-ASD, *n* = 30,876		*p*-value ^a^	HQ-traits-group,^b^ *n* = 7783		LQ-traits-group,^c^ *n* = 7783		*p*-value ^a^
Age, years (mean, *SD*)	41.2	(12.0)	50.7	(12.1)	<0.01	50.9	(12.7)	49.8	(11.4)	<0.01
Female sex (*N*, %)	124	(40.1)	18,089	(58.6)	<0.01	3534	(45.4)	5385	(69.2)	<0.01
ASD diagnosis (*N*, %)	309	(100)	0			267	(3.4)	2	(0.0)	<0.01
AQ-10 sum score^d^ (median, IQR)	6.0	(4.5–7.5)	2.0	(0.5–3.5)	<0.01	5.0	(4.0–6.0)	1.0	(0.0–1.0)	<0.01
Ethnicity (*N*, %) Eastern or Western European Mediterranean, Arabic, Black or Asian Other	275 0 <10	(89.0) (<3.2)	29,047 175 174	(94.1) (0.6) (0.6)	N.S.	7254 33 61	(93.2) (0.4) (0.8)	7392 38 29	(95.0) (0.5) (0.4)	0.03
Educational attainment (*N*, %)										
Low Middle High	47 120 90	(15.2) (38.8) (29.1)	6242 9775 10,012	(20.2) (31.7) (32.4)	0.03	2025 2578 1978	(26.0) (33.1) (25.4)	1009 2334 3202	(13.0) (30.0) (41.1)	<0.01
Employment (*N*, %)	191	(61.8)	21,502	(69.6)	0.01	5085	(65.3)	5853	(75.2)	<0.01
Gastrointestinal symptoms^e^ (*N*, %) Constipation Diarrhoea Heartburn Abdominal pain/discomfort	112 56 33 15 53	(36.2) (18.1) (10.7) (4.9) (17.2)	6446 2491 2067 963 3151	(20.9) (8.1) (6.7) (3.1) (10.2)	<0.01 <0.01 <0.01 N.S. <0.01	1919 760 629 292 936	(24.7) (9.8) (8.1) (3.8) (12.0)	1467 560 484 211 687	(18.8) (7.2) (6.2) (2.7) (8.8)	<0.01 <0.01 <0.01 <0.01 <0.01

ASD: autism spectrum disorder; IQR: interquartile range.

a Univariable analysis with Chi-square test, independent sample t-test or Mann–Whitney *U*-test.

b HQ-traits-group = 25% of participants with the highest Autism Quotient (AQ-10) sum scores.

c LQ-traits-group = 25% of participants with the lowest AQ-10 sum scores.

d AQ-10 = short version of the Autism Spectrum Quotient.

e Gastrointestinal symptoms = presence of 1 or more of these symptoms: constipation, diarrhoea, heartburn, abdominal pain/discomfort.

The first non-response analysis revealed that the 71,428 participants who were not included because they did not submit the AUTQ, were younger in age and consisted of more men compared to the 37,924 participants who submitted the AUTQ. The second non-response analysis showed that the 6726 participants who were excluded because they did not answer any of the questions in the GI-questionnaire, more frequently had an ASD diagnosis (*n* = 128; 1.9%) compared to the total included study population of 31,185 participants (*n* = 309; 1.0%). Nonetheless, the median AQ-10 sum score in the excluded 6726 participants was not different from the median AQ-10 sum score in the included study population.

### GI symptoms

The prevalence of GI symptoms (abdominal discomfort/pain, diarrhoea, constipation, and/or heartburn) was significantly higher in adults with ASD compared with adults without ASD (36% vs 21%, *p* < 0.01; Table 1). This tendency was also observed in the quartiles: 25% in the HQ-traits-group compared with 19% in the LQ-traits-group (*p* < 0.01). In adults with ASD, three out of four investigated GI symptoms were more frequently reported (*p*-values < 0.01). Subsequently, in the HQ-traits-group all four investigated GI-symptoms were more frequent, compared with the LQ-traits-group (*p*-values < 0.01). In addition, GI diseases (Crohn’s disease, ulcerative colitis, coeliac disease, gastric ulcer and/or gallstones) were not more prevalent in adults with ASD compared with adults without ASD.

### Psychological, behavioural and biological factors

In Table 2 (ASD-group vs non-ASD-group) and in Table 3 (HQ-traits-group vs LQ-traits-group), the outcomes of differences within groups (based on univariable and multivariable analyses) regarding psychological, behavioural and biological factors are presented.

**Table 2.** Psychological, behavioural and biological factors in ASD-group versus non-ASD-group.

	ASD, *n* = 309		Non-ASD, *n* = 30,876		*p*-value ^a^	Adjusted OR (95% CI)^b^
Psychological						
Psychiatric comorbidity^c^ (*N*, %)	88	(28.5)	2042	(6.6)	<0.01	5.29 (3.94–7.10)
Anxiety	75	(24.3)	1735	(5.6)	<0.01	4.98 (3.67–6.76)
Depression	49	(15.9)	812	(2.6)	<0.01	5.50 (3.81–7.92)
Stress (median, IQR) ^d^	3	(2–6)	1	(0–3)	<0.01	1.22 (1.18–1.27)
Perceived health (mean, *SD*) ^e^	2.9	(0.9)	2.6	(0.8)	<0.01	1.71 (1.47–2.00)
Behavioural						
Alcohol use, >2 glasses/day (*N*, %)	67	(21.7)	7638	(24.7)	N.S.	0.57 (0.41–0.80)
Physical activity, days/week (mean, *SD*)	4.3	(2.0)	4.4	(1.9)	N.S.	N.S.
Biological						
Body mass index (mean, *SD*) Waist circumference (cm) (mean, *SD*)	25.8 90.4	(4.9) (14.2)	25.9 90.0	(4.2) (12.4)	N.S. N.S.	N.S. N.S.
Leukocytes (10^E^9/L) (median, IQR) Neutrophils (10^E^9/L) (median, IQR) Lymphocytes (10^E^9/L) (median, IQR) Monocytes (10^E^9/L) (median, IQR) Eosinophils (10^E^9/L) (median, IQR) Basophils (10^E^9/L) (median, IQR)	6.00 3.09 1.97 0.49 0.16 0.04	(5.10–6.90) (2.60–3.84) (1.59–2.36) (0.42–0.60) (0.09–0.24) (0.03–0.06)	5.80 3.06 1.90 0.48 0.16 0.04	(4.90–6.80) (2.27–3.80) (1.57–2.31) (0.39–0.58) (0.11–0.24) (0.03–0.06)	<0.05 N.S. N.S. <0.05 N.S. N.S.	N.S. N.S. N.S. N.S. N.S. N.S.

95% CI: 95% confidence interval; ASD: autism spectrum disorder; IQR: interquartile range; OR: odds ratio.

a Univariable analysis with Chi-square test, Fisher’s exact test, independent sample *t*-test or Mann–Whitney *U*-test.

b Multivariable logistic regression, adjusted for age, sex, socioeconomic status (SES: employment and educational attainment).

c Psychiatric comorbidity = any current depressive disorder and/or any current anxiety disorder.

d Long-term difficulties inventory sum score. A higher score equals more perceived stress.

e A higher score equals worse perceived general health.

**Table 3.** Psychological, behavioural and biological factors in HQ-traits-group versus LQ-traits-group.

	HQ-traits-group,^a^ *n* = 7783		LQ-traits-group,^b^ *n* = 7783		*p*-value ^c^	Adjusted OR (95% CI)^d^
Psychological Psychiatric comorbidity^e^ (*N*, %) Anxiety (*N*, %) Depression (*N*, %)	855 714 396	(11.0) (9.2) (5.1)	316 269 107	(4.1) (3.5) (1.4)	<0.01 <0.01 <0.01	3.27 (2.83–3.79) 3.20 (2.74–3.75) 3.92 (3.10–4.95)
Stress (median, IQR)^f^	2.0	(0.0–3.0)	1.0	(0.0–3.0)	<0.01	1.15 (1.13–1.17)
Perceived health (mean, *SD*)^g^	2.8	(0.8)	2.5	(0.8)	<0.01	1.48 (1.41–1.55)
Behavioural						
Alcohol use, >2 glasses/day (*N*, %)	2088	(26.8)	1752	(22.5)	<0.01	N.S.
Physical activity, days/week (mean, *SD*)	4.2	(1.9)	4.5	(1.9)	<0.01	0.95 (0.93–0.97)
Biological						
Body mass index (mean, *SD*) Waist circumference (cm) (mean, *SD*)	26.1 91.5	(4.3) (12.6)	25.6 88.4	(4.2) (12.1)	<0.01 <0.01	N.S N.S.
Leukocytes (10^E^9/L) (median, IQR) Neutrophils (10^E^9/L) (median, IQR) Lymphocytes (10^E^9/L) (median, IQR) Monocytes (10^E^9/L) (median, IQR) Eosinophils (10^E^9/L) (median, IQR) Basophils (10^E^9/L) (median, IQR) CRP (median, IQR)	5.80 3.09 1.90 0.49 0.16 0.04 1.1	(5.00–6.90) (2.50–3.83) (1.56–2.31) (0.40–0.59) (0.11–0.24) (0.03–0.06) (0.6–3.0)	5.70 3.04 1.90 0.47 0.16 0.04 1.0	(4.90–6.80) (2.43–3.78) (1.57–2.29) (0.39–0.56) (0.11–0.24) (0.03–0.06) (0.5–2.2)	<0.01 <0.01 N.S. <0.01 <0.01 N.S. N.S.	N.S. N.S. N.S. N.S. N.S. N.S. N.S.

95% CI: 95% confidence interval; CRP: C-reactive protein; IQR: interquartile range; OR: odds ratio; *SD*: standard deviation.

a HQ-traits-group = 25% of participants with the highest Autism Quotient (AQ-10) sum scores.

b LQ-traits-group = 25% of participants with the lowest AQ-10 sum scores.

c Univariable analysis with chi-square test, Fisher’s exact test, independent sample t-test or Mann–Whitney U test.

d Multivariable logistic regression, adjusted for age, sex, socioeconomic status (SES: employment and educational attainment).

e Psychiatric comorbidity = any current depressive disorder and/or any current anxiety disorder.

f Long-term difficulties inventory sum score. A higher score equals more perceived stress.

g A higher score equals worse perceived general health.

Psychiatric comorbidity (anxiety and/or depression) was more common in the ASD-group (OR 5.29, 95% CI 3.94–7.10). Adults with ASD reported higher stress levels (OR 1.22, 95% CI 1.18–1.27) and worse perceived health (OR 1.71, 95% CI 1.47–2.00; as a higher score resembles worse perceived health), compared with adults without ASD. When comparing the HQ-traits-group and LQ-traits-group, the same differences were observed: psychiatric comorbidity was more prevalent in the HQ-traits-group (OR 3.27, 95% CI 2.83–3.79), stress levels were higher (OR 1.15, 95% CI 1.13–1.17), and perceived health was worse (OR 1.48, 95% CI 1.41–1.55).

Regarding behavioural factors, the level of physical activity and prevalence of alcohol use of more than two glasses per day in adults with ASD did not differ from adults without ASD. Multivariable analyses, correcting for differences in age, sex and socioeconomic status, also revealed no difference in the prevalence of alcohol use between the HQ-traits-group and LQ-traits-group. These analyses did demonstrate that adults in the HQ-traits-group were less physically active (OR 0.95, 95% CI 0.93–0.97) than adults in the LQ-traits-group.

Multivariable analyses of biological measurements showed that mean BMI and waist circumference were not different between both the ASD-group and non-ASD-group and the HQ-traits-group and LQ-traits-group. Similar approaches of immune parameters revealed no differences between the ASD-group and non-ASD-group and the HQ-traits-group and LQ-traits-group regarding the total leukocyte and leukocyte-subset counts as well as CRP levels.

### Factors associated with gastrointestinal symptoms

Assessing GI symptoms in the distinct groups and associated factors (Table 4), we observed that GI-symptoms were associated with all investigated psychological variables (psychiatric comorbidity, stress and perceived health) in the ASD-, non-ASD-, HQ-traits- and LQ-traits-groups. In the ASD-group, GI symptoms were not associated with behavioural variables (alcohol use and physical activity). In the HQ-traits-group, being less physically active was associated with GI symptoms (OR 0.95, 95% CI 0.92–0.98). No association was found between alcohol use and GI symptoms in the HQ-traits-group. Most biological variables (BMI, waist circumference, eosinophils, monocytes and basophils) were not associated with GI symptoms in any of the investigated groups. However, in the HQ-traits-group as well as in the non-ASD-group, GI symptoms were positively associated with total leukocyte, lymphocyte and neutrophil counts.

**Table 4.** Multivariable logistic regression with GI symptoms as outcome, adjusted for confounders^a^.

	ASD, *n* = 309	Non-ASD, *n* = 30,876	HQ-traits-group,^b^ *n* = 7783	LQ-traits-group,^c^ *n* = 7783
	GI symptoms^d^ (OR, 95% CI)^e^	GI symptoms^d^ (OR, 95% CI)^e^	GI symptoms^d^ (OR, 95% CI)^e^	GI symptoms^d^ (OR, 95% CI)^e^
Psychological				
Psychiatric comorbidity^f^ Stress^g^ Perceived health^h^	2.78 (1.51–5.01)* 1.15 (1.06–1.26)*2.32 (1.62–3.34)*	2.72 (2.46–3.01)* 1.20 (1.18–1.21)*2.07 (1.99–2.16)*	2.68 (2.27–3.15)* 1.18 (1.15–1.21)*2.16 (1.98–2.35)*	2.45 (1.91–3.13)* 1.20 (1.16–1.23)*2.06 (1.89–2.24)*
Behavioural				
Alcohol use, >2 glasses/day	0.77 (0.38–1.59)	1.07 (0.99–1.16)	1.04 (0.90–1.21)	1.18 (1.00–1.39)
Physical activity	0.92 (0.80–1.05)	0.96 (0.95–0.98)*	0.95 (0.92–0.98)*	0.96 (0.93–1.00)
Biological				
Body mass index Waist circumference	1.01 (0.96–1.07) 1.00 (0.98–1.02)	1.01 (1.00–1.02) 1.01 (1.00–1.01)	1.01 (1.00–1.02) 1.01 (1.00–1.01)	1.02 (1.00–1.03) 1.01 (1.00–1.01)
Leukocytes	0.99 (0.85–1.14)	1.04 (1.02–1.06)*	1.05 (1.01–1.08)*	1.04 (1.00–1.08)
Neutrophils Lymphocytes Monocytes	1.06 (0.84–1.34) 0.76 (0.49–1.19) 0.36 (0.05–2.85)	1.07 (1.04–1.09)* 1.08 (1.03–1.13)* 1.23 (0.99–1.52)	1.07 (1.02–1.12)* 1.11 (1.01–1.22)* 0.94 (0.63–1.40)	1.07 (1.01–1.12)* 0.98 (0.89–1.09) 1.25 (0.80–1.96)
Eosinophils	2.87 (0.48–17.22)	0.96 (0.76–1.21)	1.01 (0.65–1.57)	1.43 (0.92–2.20)
Basophils	0.80 (0.00–597051.25)	2.09 (0.52–8.44)	6.86 (0.51–92.77)	1.34 (0.08–23.88)
CRP	–	1.16 (0.57–2.36)	1.24 (0.31–5.04)	0.68 (0.13–3.66)

95% CI: 95% confidence interval; ASD: autism spectrum disorder; CRP: C-reactive protein; OR: odds ratio.

a In each of the four groups (ASD-, non-ASD-, HQ-traits- and LQ-traits-groups), separate multivariable logistic regression models were performed with the presence of 1 or more GI symptom(s) as dependent outcome measure. For each of the independent psychological, behavioural and biological variables a separate multivariable logistic regression model was performed, including adjustment for confounders (age, sex, employment and educational attainment). Thus, the groups were not compared in these analyses.

b HQ-traits-group = 25% of participants with the highest Autism Quotient (AQ-10) sum scores.

c LQ-traits-group = 25% of participants with the lowest AQ-10 sum scores.

d Gastrointestinal symptoms = presence of 1 or more of these symptoms: constipation, diarrhoea, heartburn, abdominal pain/discomfort.

e Statistically significant odds ratios are marked: *.

f Psychiatric comorbidity = any current depressive disorder and/or any current anxiety disorder.

g Long-term difficulties inventory sum score. A higher score equals more perceived stress.

h A higher score equals worse perceived general health.

Interaction term analyses demonstrated that there were no interactions between the variable ‘ASD or non-ASD’ and the included psychological, behavioural, and biological factors. The only significant interaction was between the variable ‘HQ-traits- or LQ-traits-group’ and lymphocyte counts (OR 1.16, 95% CI 1.01–1.33).

## Discussion

Our results demonstrated an elevated risk for the GI symptoms abdominal discomfort/pain, diarrhoea, constipation and/or heartburn in adults with ASD compared to adults without ASD. This increased risk was also found in adults with higher levels of autistic traits compared to adults with lower levels. In both adults with ASD and adults with higher levels of autistic traits, GI symptoms were strongly associated with psychiatric comorbidity (anxiety and/or depressive disorder), increased perceived psychological stress levels and worse perceived health. In adults with higher levels of autistic traits, there was a moderate association between GI symptoms and less physical activity and increased leukocyte, lymphocyte and neutrophil counts.

With regard to psychological factors, we found no previous studies specifically investigating the association between GI symptoms in adults with ASD or autistic traits and stress or psychiatric comorbidities. Nonetheless, interactions between autistic traits and several psychological parameters have already been acknowledged (Amos et al., 2019; Green & Ben-Sasson, 2010), for example, sensory over-responsivity, which could lead to stress and anxiety. Through the gut-brain axis, psychological factors such as stress and anxiety can cause alterations in the GI tract (e.g. bowel movement and gut microbiota) and vice versa (Chidambaram et al., 2020). Such GI alterations are related to GI symptoms. On a biological basis, this bilateral interaction between the physique and the psyche in the gut-brain axis supports the associations we observed between GI symptoms and psychiatric comorbidity and stress in autistic adults. The current study also showed that in adults with ASD and in adults with higher levels of autistic traits, worse perceived health was associated with GI symptoms. This result could be explained by the relation between worse perceived health and stress, as found in autistic adults (Moseley et al., 2021).

Regarding investigated behavioural factors, alcohol use of more than two glasses per day was not associated with GI symptoms in our adults with ASD or autistic traits. Previous studies demonstrated that alcohol (ab)use is less prevalent in adults with ASD (Croen et al., 2015; Tolchard & Stuhlmiller, 2018; Vohra et al., 2017; Weir et al., 2021), as also observed in our multivariable analysis. When it comes to physical activity, previous literature has pointed out that children and adults with ASD on average perform less physical exercise (Hillier et al., 2020; McCoy et al., 2016; Memari et al., 2015). Moreover, the current study displayed that being less physically active was associated with GI symptoms in adults with higher levels of autistic traits. Therefore, it is advisable to take physical activity into account when assessing GI problems in autistic adults.

With respect to biological factors, BMI is associated with GI symptoms in the general population (Eslick, 2012) and weight problems are an increasing epidemic in people with ASD (Croen et al., 2015; Hand et al., 2020; Li et al., 2020). In our study, mean BMI and waist circumference did not differ between adults with and without ASD, and both were not associated with GI symptoms. Regarding immunological factors, we investigated leukocyte and subtype counts because stress is a mediator in GI symptoms and stress hormones (such as cortisol) are known to regulate these leukocyte counts (De Palma et al., 2014; Dhabhar et al., 2012). Therefore, we hypothesised that altered immunological blood markers might help to understand the correlation between chronic exposure to stress in individuals with ASD and the occurrence of GI symptoms (Baron et al., 2006; Bishop-Fitzpatrick et al., 2015; Hirvikoski & Blomqvist, 2015). Our study demonstrated a moderate association between immunological factors, in particular lymphocyte and neutrophil counts, and GI symptoms in adults with higher levels of autistic traits. In adults with lower levels of autistic traits, neutrophil but not lymphocyte counts were found to be associated with GI symptoms. Thus, we cannot conclude that these total leukocyte and subtype counts clearly highlight the relationship between stress and GI symptoms in our target population.

In summary, our findings suggest that stress, psychiatric comorbidities, perceived health and physical activity are important factors to integrate in the assessment of GI symptoms in adults with ASD or autistic traits. It should be noted that our study population included adults that were able to provide self-reported AQ-10 answers. Therefore, it is estimated that this study population mostly represents adults with lower support needs. It is hypothesised that future inclusion of adults across a wider range of intellectual abilities would result in similar or magnified findings compared to our study since intellectual disability increases the risk for GI disorders in adults with ASD (Bishop-Fitzpatrick & Rubenstein, 2019; Gilmore et al., 2021). It is also expected that investigating a study population including more adults with ASD would result in larger significant findings regarding factors associated with GI symptoms in these adults with ASD because a larger sample size leads to more precision.

### Strengths

The main strength of this study is the large sample size of a prospective general cohort, offering a wide range of basic, psychological, behavioural and biological variables in more than 31,000 adults. The approximately 1% (*n* = 309) of adults reporting an ASD diagnosis in our study sample matches the worldwide prevalence of 1%–2% (Baio et al., 2018; Dietz et al., 2020). This makes our study population a representative sample in terms of autism prevalence. Another asset to this study is the assessment of stress levels by perceived psychological stress using questionnaires and a laboratory approach by immunological blood markers. Furthermore, the combination of performing the same analyses in both adults with ASD and adults with autistic traits is relevant to include in research because of the prevalence of undiagnosed ASD cases (Baron-Cohen et al., 2009) and the correlations between autistic traits and GI symptoms (Amos et al., 2019; Williams & Gotham, 2022). A final strength of this study is the involvement of the autistic community in defining the research question, including relevant variables and interpreting the results (through advice of autistic team-members).

### Limitations

Since this study had a cross-sectional design, it was not possible to examine temporality. Another limitation of our study is the fact that data from the GI questionnaire and the autism questionnaire were not available at the same moment in time. This resulted in a mean gap of 4 years between data about the ASD diagnosis and AQ-10 scores, and psychological, behavioural, biological and GI data. Nonetheless, since ASD is considered as a lifelong diagnosis, this time gap was not likely to interfere with our results in the ASD- and non-ASD-groups. Broadbent et al. (2013) concluded that the AQ-10 test–retest reliability, with a time interval of 6 to 12 months, was satisfactory. As it is known that food selectivity and insufficient oral intake can affect the intestinal microbiome in children with ASD (Bandini et al., 2010; Murtaza et al., 2017), it is a limitation of our study that dietary variables were not included. Our study was limited to self-report regarding the presence of an ASD diagnosis. However, this uncertainty was partly countered by a median AQ-10 sum score of 6.0 in adults with ASD, compared to 2.0 in the adults without ASD. It should also be taken into account that GI symptoms were based on self-report (ROME-III questions). However, Lefter et al. (2019) advised to use ROME questionnaires to assess GI problems in ASD. Since people were not eligible for inclusion in the Lifelines Cohort if they did not have the ability to fill in self-report surveys, our study sample does not represent those autistic adults with cognitive disabilities or other types of disabilities impacting self-report. Therefore, the results from our study cannot be generalised to all types of autistic adults but are limited to autistic adults with the ability to fill in self-report surveys.

### Implications

Both healthcare providers and autistic adults should be aware that psychological and behavioural factors are associated with GI symptoms in autistic adults. It would be beneficial to people with ASD if this knowledge results in more integrated psychological, behavioural and somatic care. Since we found that adults with autistic traits exhibit more psychological and behavioural risk factors, it could be useful to consider autistic traits in patients with poorly understood GI complaints. After exclusion of a somatic cause of GI problems, stress, perceived health, psychiatric comorbidity and physical activity should be evaluated by clinicians. Moreover, health education focusing on psychosomatic interactions could lead to better adherence of treatment or preventive measures. Future research should address the optimalisation of prevention and treatment of GI symptoms in autistic adults. Finally, associations between GI symptoms and increased leukocyte (subtype) counts in autistic adults should be investigated in more depth to clarify their value for clinical practice.

## Conclusion

Our study demonstrates that not only adults with ASD but also adults with higher levels of autistic traits are at increased risk for GI symptoms. In addition, in both adults with ASD and adults with higher levels of autistic traits, GI symptoms are strongly associated with higher stress levels, psychiatric comorbidity and worse perceived health. In adults with the highest levels of autistic traits, GI symptoms are also moderately associated with less physical activity. Together, these results suggest that assessment of GI symptoms in adults with ASD or autistic traits might benefit from an integrated psychological and behavioural approach, next to the somatic assessment. Future research could focus on optimising clinical practice regarding prevention and treatment of GI symptoms in autistic adults.

## References

[bibr1-13623613231155324] Allison C. Auyeung B. Baron-Cohen S. (2012). Toward brief ‘Red Flags’ for autism screening: The Short Autism Spectrum Quotient and the Short Quantitative Checklist for autism in toddlers in 1,000 cases and 3,000 controls [corrected]. Journal of the American Academy of Child and Adolescent Psychiatry, 51(2), 202–212.e7. 10.1016/j.jaac.2011.11.003 22265366

[bibr2-13623613231155324] Amos G. A. Byrne G. Chouinard P. A. Godber T. (2019). Autism traits, sensory over-responsivity, anxiety, and stress: A test of explanatory models. Journal of Autism and Developmental Disorders, 49(1), 98–112. 10.1007/s10803-018-3695-6 30043351

[bibr3-13623613231155324] Ashwood K. L. Gillan N. Horder J. Hayward H. Woodhouse E. McEwen F. S. Findon J. Eklund H. Spain D. Wilson C. E. Cadman T. Young S. Stoencheva V. Murphy C. M. Robertson D. Charman T. Bolton P. Glaser K. Asherson P. Murphy D. G. (2016). Predicting the diagnosis of autism in adults using the Autism-Spectrum Quotient (AQ) Questionnaire. Psychological Medicine, 46(12), 2595–2604. 10.1017/S0033291716001082 27353452 PMC4988267

[bibr4-13623613231155324] Baio J. Wiggins L. Christensen D. L. Maenner M. J. Daniels J. Warren Z. Kurzius-Spencer M. Zahorodny W. Robinson Rosenberg C. White T. Durkin M. S. Imm P. Nikolaou L. Yeargin-Allsopp M. Lee L. C. Harrington R. Lopez M. Fitzgerald R. T. Hewitt A. . . .Dowling N. F . (2018). Prevalence of autism spectrum disorder among children aged 8 years - autism and developmental disabilities monitoring network, 11 sites, United States, 2014. Morbidity and Mortality Weekly Report, 67(6), 1–23. 10.15585/mmwr.ss6706a1 29701730 PMC5919599

[bibr5-13623613231155324] Bandini L. G. Anderson S. E. Curtin C. Cermak S. Evans E. W. Scampini R. Maslin M. Must A. (2010). Food selectivity in children with autism spectrum disorders and typically developing children. The Journal of Pediatrics, 157(2), 259–264. 10.1016/j.jpeds.2010.02.013 20362301 PMC2936505

[bibr6-13623613231155324] Baron M. G. Groden J. Groden G. Lipsitt L. P. (2006). Stress and coping in autism. Oxford University Press.

[bibr7-13623613231155324] Baron-Cohen S. Scott F. J. Allison C. Williams J. Bolton P. Matthews F. E. Brayne C. (2009). Prevalence of autism-spectrum conditions: UK school-based population study. British Journal of Psychiatry, 194(6), 500–509. 10.1192/bjp.bp.108.059345 19478287

[bibr8-13623613231155324] Bilski J. Mazur-Bialy A. Magierowski M. Kwiecien S. Wojcik D. Ptak-Belowska A. Surmiak M. Targosz A. Magierowska K. Brzozowski T. (2018). Exploiting significance of physical exercise in prevention of gastrointestinal disorders. Current Pharmaceutical Design, 24(18), 1916–1925. 10.2174/1381612824666180522103759 29788876

[bibr9-13623613231155324] Bishop-Fitzpatrick L. Mazefsky C. A. Minshew N. J. Eack S. M. (2015). The relationship between stress and social functioning in adults with autism spectrum disorder and without intellectual disability. Autism Research, 8(2), 164–173. 10.1002/aur.1433 25524571 PMC4412754

[bibr10-13623613231155324] Bishop-Fitzpatrick L. Rubenstein E. (2019). The physical and mental health of middle aged and older adults on the autism spectrum and the impact of intellectual disability. Research in Autism Spectrum Disorders, 63, 34–41. 10.1016/j.rasd.2019.01.001 31768189 PMC6876625

[bibr11-13623613231155324] Booth T. Murray A. L. McKenzie K. Kuenssberg R. O’Donnell M. Burnett H. (2013). Brief report: An evaluation of the AQ-10 as a brief screening instrument for ASD in adults. Journal of Autism and Developmental Disorders, 43(12), 2997–3000. 10.1007/s10803-013-1844-5 23640304

[bibr12-13623613231155324] Broadbent J. Galic I. Stokes M. A. (2013). Validation of autism spectrum quotient adult version in an Australian sample. Autism Research and Treatment, 2013, 984205. 10.1155/2013/984205 PMC3665170 23762552

[bibr13-13623613231155324] Bytzer P. Howell S. Leemon M. Young L. J. Jones M. P. Talley N. J. (2001). Low socioeconomic class is a risk factor for upper and lower gastrointestinal symptoms: A population based study in 15 000 Australian adults. Gut, 49(1), 66–72. 10.1136/gut.49.1.66 11413112 PMC1728377

[bibr14-13623613231155324] Cacioppo J. T. Tassinary L. G. Berntson G. (2007). The handbook of psychophysiology (3rd ed.). Cambridge University Press.

[bibr15-13623613231155324] Chen G. Haber P. S. (2021). Gastrointestinal disorders related to alcohol and other drug use. In el-Guebaly N. Carrà G. Galanter M. Baldacchino A. M. (Eds.), Textbook of addiction treatment. Springer. 10.1007/978-3-030-36391-8_76

[bibr16-13623613231155324] Chidambaram S. B. Tuladhar S. Bhat A. Mahalakshmi A. M. Ray B. Essa M. M. Bishir M. Bolla S. R. Nanjaiah N. D. Guillemin G. J. Qoronfleh M. W. (2020). Autism and gut-brain axis: Role of probiotics. Advances in Neurobiology, 24, 587–600. 10.1007/978-3-030-30402-7_21 32006375

[bibr17-13623613231155324] Croen L. A. Zerbo O. Qian Y. Massolo M. L. Rich S. Sidney S. Kripke C. (2015). The health status of adults on the autism spectrum. Autism, 19(7), 814–823. 10.1177/1362361315577517 25911091

[bibr18-13623613231155324] Del Giudice M. Gangestad S. W. (2018). Rethinking IL-6 and CRP: Why they are more than inflammatory biomarkers, and why it matters. Brain, Behavior, and Immunity, 70, 61–75. 10.1016/j.bbi.2018.02.013 29499302

[bibr19-13623613231155324] De Palma G. Collins S. M. Bercik P. Verdu E. F. (2014). The microbiota-gut-brain axis in gastrointestinal disorders: Stressed bugs, stressed brain or both? The Journal of Physiology, 592(14), 2989–2997. 10.1113/jphysiol.2014.273995 24756641 PMC4214655

[bibr20-13623613231155324] Dhabhar F. S. Malarkey W. B. Neri E. McEwen B. S. (2012). Stress-induced redistribution of immune cells – From barracks to boulevards to battlefields: A tale of three hormones – Curt Richter Award winner. Psychoneuroendocrinology, 37(9), 1345–1368. 10.1016/j.psyneuen.2012.05.008 22727761 PMC3412918

[bibr21-13623613231155324] Dietz P. M. Rose C. E. McArthur D. Maenner M. (2020). National and state estimates of adults with autism spectrum disorder. Journal of Autism and Developmental Disorders, 50(12), 4258–4266. 10.1007/s10803-020-04494-4 32390121 PMC9128411

[bibr22-13623613231155324] Dijkstra-de Neijs L. Leenen P. J. M. Hays J. P. Van der Valk E. S. Kraaij R. Van Rossum E. F. C. Ester W. A. (2020). Biological consequences of psychological distress in caregivers of children with autism spectrum disorder and its potential relevance to other chronic diseases including cancer. Current Epidemiology Reports, 7, 139–148. 10.1007/s40471-020-00237-2

[bibr23-13623613231155324] Emerenziani S. Guarino M. Trillo Asensio L. M. Altomare A. Ribolsi M. Balestrieri P. Cicala M. (2019). Role of overweight and obesity in gastrointestinal disease. Nutrients, 12(1), 111. 10.3390/nu12010111 31906216 PMC7019431

[bibr24-13623613231155324] Eslick G. D. (2012). Gastrointestinal symptoms and obesity: A meta-analysis. Obesity Reviews, 13(5), 469–479. 10.1111/j.1467-789X.2011.00969.x 22188520

[bibr25-13623613231155324] Ferguson B. J. Marler S. Altstein L. L. Lee E. B. Akers J. Sohl K. McLaughlin A. Hartnett K. Kille B. Mazurek M. Macklin E. A. McDonnell E. Barstow M. Bauman M. L. Margolis K. G. Veenstra-VanderWeele J. Beversdorf D. Q. (2017). Psychophysiological associations with gastrointestinal symptomatology in autism spectrum disorder. Autism Research, 10(2), 276–288. 10.1002/aur.1646 27321113 PMC5526214

[bibr26-13623613231155324] Ferguson B. J. Marler S. Altstein L. L. Lee E. B. Mazurek M. O. McLaughlin A. Macklin E. A. McDonnell E. Davis D. J. Belenchia A. M. Gillespie C. H. Peterson C. A. Bauman M. L. Margolis K. G. Veenstra-VanderWeele J. Beversdorf D. Q. (2016). Associations between cytokines, endocrine stress response, and gastrointestinal symptoms in autism spectrum disorder. Brain, Behavior, and Immunity, 58, 57–62. 10.1016/j.bbi.2016.05.009 27181180 PMC5526212

[bibr27-13623613231155324] Foxx-Orenstein A. E. (2010). Gastrointestinal symptoms and diseases related to obesity: An overview. Gastroenterology Clinics of North America, 39(1), 23–37. 10.1016/j.gtc.2009.12.006 20202576

[bibr28-13623613231155324] Gilmore D. Harris L. Longo A. Hand B. N. (2021). Health status of Medicare-enrolled autistic older adults with and without co-occurring intellectual disability: An analysis of inpatient and institutional outpatient medical claims. Autism, 25(1), 266–274. 10.1177/1362361320955109 32907348

[bibr29-13623613231155324] Green S. A. Ben-Sasson A. (2010). Anxiety disorders and sensory over-responsivity in children with autism spectrum disorders: Is there a causal relationship? Journal of Autism and Developmental Disorders, 40(12), 1495–1504. 10.1007/s10803-010-1007-x 20383658 PMC2980623

[bibr30-13623613231155324] Hand B. N. Angell A. M. Harris L. Carpenter L. A. (2020). Prevalence of physical and mental health conditions in Medicare-enrolled, autistic older adults. Autism, 24(3), 755–764. 10.1177/1362361319890793 31773968 PMC7433648

[bibr31-13623613231155324] Harris H. A. Micali N. Moll H. A. van Berckelaer-Onnes I. Hillegers M. Jansen P. W. (2021). The role of food selectivity in the association between child autistic traits and constipation. International Journal of Eating Disorders, 54(6), 981–985. 10.1002/eat.23485 33594728 PMC8248436

[bibr32-13623613231155324] Hillier A. Buckingham A. Schena D. II . (2020). Physical activity among adults with autism: Participation, attitudes, and barriers. Perceptual and Motor Skills, 127(5), 874–890. 10.1177/0031512520927560 32443953

[bibr33-13623613231155324] Hirvikoski T. Blomqvist M. (2015). High self-perceived stress and poor coping in intellectually able adults with autism spectrum disorder. Autism, 19(6), 752–757. 10.1177/1362361314543530 25073750

[bibr34-13623613231155324] Holingue C. Newill C. Lee L. C. Pasricha P. J. Daniele Fallin M. (2018). Gastrointestinal symptoms in autism spectrum disorder: A review of the literature on ascertainment and prevalence. Autism Research, 11(1), 24–36. 10.1002/aur.1854 28856868 PMC5773354

[bibr35-13623613231155324] Hollins S. L. Hodgson D. M. (2019). Stress, microbiota, and immunity. Current Opinion in Behavioral Sciences, 28, 66–71. 10.1016/j.cobeha.2019.01.015

[bibr36-13623613231155324] Knutsson A. Bøggild H. (2010). Gastrointestinal disorders among shift workers. Scandinavian Journal of Work, Environment & Health, 36(2), 85–95. 10.5271/sjweh.2897 20101379

[bibr37-13623613231155324] Lai M. C. Lombardo M. V. Baron-Cohen S. (2014). Autism. The Lancet, 383(9920), 896–910. 10.1016/S0140-6736(13)61539-1 24074734

[bibr38-13623613231155324] Leader G. Barrett A. Ferrari C. Casburn M. Maher L. Naughton K. Arndt S. Mannion A. (2021). Quality of life, gastrointestinal symptoms, sleep problems, social support, and social functioning in adults with autism spectrum disorder. Research in Developmental Disabilities, 112, 103915. 10.1016/j.ridd.2021.103915 33676088

[bibr39-13623613231155324] Lee C. G. Lee J. K. Kang Y. S. Shin S. Kim J. H. Lim Y. J. Koh M. S. Lee J. H. Kang H. W. (2015). Visceral abdominal obesity is associated with an increased risk of irritable bowel syndrome. The American Journal of Gastroenterology, 110(2), 310–319. 10.1038/ajg.2014.422 25583325

[bibr40-13623613231155324] Lefter R. Ciobica A. Timofte D. Stanciu C. Trifan A. (2019). A descriptive review on the prevalence of gastrointestinal disturbances and their multiple associations in autism spectrum disorder. Medicina, 56(1), 11. 10.3390/medicina56010011 31892195 PMC7023358

[bibr41-13623613231155324] Li Y. J. Xie X. N. Lei X. Li Y. M. Lei X. (2020). Global prevalence of obesity, overweight and underweight in children, adolescents and adults with autism spectrum disorder, attention-deficit hyperactivity disorder: A systematic review and meta-analysis. Obesity Reviews, 21(12), e13123. 10.1111/obr.13123 32783349

[bibr42-13623613231155324] Longstreth G. F. Thompson W. G. Chey W. D. Houghton L. A. Mearin F. Spiller R. C. (2006). Functional bowel disorders. Gastroenterology, 130(5), 1480–1491. 10.1053/j.gastro.2005.11.061 16678561

[bibr43-13623613231155324] Lundin A. Kosidou K. Dalman C. (2019). Measuring autism traits in the adult general population with the brief Autism-Spectrum Quotient, AQ-10: Findings from the Stockholm Public Health Cohort. Journal of Autism and Developmental Disorders, 49(2), 773–780. 10.1007/s10803-018-3749-9 30244391

[bibr44-13623613231155324] Mazurek M. O. Vasa R. A. Kalb L. G. Kanne S. M. Rosenberg D. Keefer A. Murray D. S. Freedman B. Lowery L. A. (2013). Anxiety, sensory over-responsivity, and gastrointestinal problems in children with autism spectrum disorders. Journal of Abnormal Child Psychology, 41(1), 165–176. 10.1007/s10802-012-9668-x 22850932

[bibr45-13623613231155324] McCoy S. M. Jakicic J. M. Gibbs B. B. (2016). Comparison of obesity, physical activity, and sedentary behaviors between adolescents with autism spectrum disorders and without. Journal of Autism and Developmental Disorders, 46(7), 2317–2326. 10.1007/s10803-016-2762-0 26936162

[bibr46-13623613231155324] McElhanon B. O. McCracken C. Karpen S. Sharp W. G. (2014). Gastrointestinal symptoms in autism spectrum disorder: A meta-analysis. Pediatrics, 133(5), 872–883. 10.1542/peds.2013-3995 24777214

[bibr47-13623613231155324] Memari A. H. Panahi N. Ranjbar E. Moshayedi P. Shafiei M. Kordi R. Ziaee V. (2015). Children with autism spectrum disorder and patterns of participation in daily physical and play activities. Neurology Research International, 2015, 531906. 10.1155/2015/531906 PMC4485548 26171247

[bibr48-13623613231155324] Moseley R. L. Turner-Cobb J. M. Spahr C. M. Shields G. S. Slavich G. M. (2021). Lifetime and perceived stress, social support, loneliness, and health in autistic adults. Health Psychology, 40(8), 556–568. 10.1037/hea0001108 34618502 PMC8513810

[bibr49-13623613231155324] Murtaza N. Ó. Cuív P. Morrison M. (2017). Diet and the microbiome. Gastroenterology Clinics of North America, 46(1), 49–60. 10.1016/j.gtc.2016.09.005 28164852

[bibr50-13623613231155324] Palsson O. S. Whitehead W. E. van Tilburg M. A. Chang L. Chey W. Crowell M. D. Keefer L. Lembo A. J. Parkman H. P. Rao S. S. Sperber A. Spiegel B. Tack J. Vanner S. Walker L. S. Whorwell P. Yang Y. (2016). Rome IV Diagnostic Questionnaires and tables for investigators and clinicians. Gastroenterology, 150(6), 1481–1491. 10.1053/j.gastro.2016.02.014 27144634

[bibr51-13623613231155324] Rausch C. van Zon S. Liang Y. Laflamme L. Möller J. de Rooij S. E. Bültmann U. (2022). Geriatric syndromes and incident chronic health conditions among 9094 older community-dwellers: Findings from the lifelines cohort study. Journal of the American Medical Directors Association, 23(1), 54–59.e2. 10.1016/j.jamda.2021.02.030 33798484

[bibr52-13623613231155324] Scholtens S. Smidt N. Swertz M. A. Bakker S. J. Dotinga A. Vonk J. M. van Dijk F. van Zon S. K. Wijmenga C. Wolffenbuttel B. H. Stolk R. P. (2015). Cohort profile: LifeLines, a three-generation cohort study and biobank. International Journal of Epidemiology, 44(4), 1172–1180. 10.1093/ije/dyu229 25502107

[bibr53-13623613231155324] Sizoo B. B. Horwitz E. H. Teunisse J. P. Kan C. C. Vissers C. Forceville E. Van Voorst A. Geurts H. M. (2015). Predictive validity of self-report questionnaires in the assessment of autism spectrum disorders in adults. Autism, 19(7), 842–849. 10.1177/1362361315589869 26088060

[bibr54-13623613231155324] Tafet G. E. Nemeroff C. B. (2016). The links between stress and depression: Psychoneuroendocrinological, genetic, and environmental interactions. The Journal of Neuropsychiatry and Clinical Neurosciences, 28(2), 77–88. 10.1176/appi.neuropsych.15030053 26548654

[bibr55-13623613231155324] Thompson W. G. Drossman D. A. Talley N. (2006). Rome III diagnostic questionnaire for the adult functional GI disorders (including alarm questions) and scoring algorithm. In Drossman D. A. Cprazzoaro E. Delvaux M. Spiller R. C. Talley N. J. Thompson W. G. Whitehead W. E. (Eds.), Rome III: The functional gastrointestinal disorders (pp. 917–951). Degnon Associates, Inc.

[bibr56-13623613231155324] Tolchard B. Stuhlmiller C. (2018). Chronic health and lifestyle problems for people diagnosed with autism in a student-led clinic. Advances in Autism, 4(2), 66–72. 10.1108/AIA-01-2018-0002

[bibr57-13623613231155324] Vohra R. Madhavan S. Sambamoorthi U. (2017). Comorbidity prevalence, healthcare utilization, and expenditures of Medicaid enrolled adults with autism spectrum disorders. Autism, 21(8), 995–1009. 10.1177/1362361316665222 27875247 PMC5517354

[bibr58-13623613231155324] Warrier V. Greenberg D. M. Weir E. Buckingham C. Smith P. Lai M. C. Allison C. Baron-Cohen S. (2020). Elevated rates of autism, other neurodevelopmental and psychiatric diagnoses, and autistic traits in transgender and gender-diverse individuals. Nature Communications, 11(1), 3959. 10.1038/s41467-020-17794-1 PMC7415151 32770077

[bibr59-13623613231155324] Weir E. Allison C. Warrier V. Baron-Cohen S. (2021). Increased prevalence of non-communicable physical health conditions among autistic adults. Autism, 25(3), 681–694. 10.1177/1362361320953652 32907337 PMC7610707

[bibr60-13623613231155324] Williams Z. J. Gotham K. O. (2022). Current and lifetime somatic symptom burden among transition-aged autistic young adults. Autism Research, 15, 761–770. 10.1002/aur.2671 35019241 PMC9115676

[bibr61-13623613231155324] Wouters H. van Zeventer I. A. van der Klauw M. M. Wolffenbuttel B. Huls G. (2020). Association between peripheral blood cell count abnormalities and health-related quality of life in the general population. Hemasphere, 5(1), e503. 10.1097/HS9.0000000000000503 PMC7755519 33364549

